# Letter regarding “Comparison of lung ultrasound, chest radiographs, C‐reactive protein, and clinical findings in dogs treated for aspiration pneumonia”

**DOI:** 10.1111/jvim.16557

**Published:** 2022-09-30

**Authors:** Gregory R. Lisciandro

**Affiliations:** ^1^ Hill Country Veterinary Specialists Spicewood Texas USA; ^2^ FASTVet.com Spicewood Texas USA

Dear Editor,

I read with interest the recent publication entitled “Comparison of lung ultrasound, chest radiographs, C‐reactive protein, and clinical findings in dogs treated for aspiration pneumonia.”^1^ This study was presented in 2019 at the European Veterinary Emergency and Critical Care Symposium (EVECCS) titled “Comparison of thoracic point of care ultrasound and radiographic findings as well as clinical evolution and C Reactive Protein concentrations in dogs treated for aspiration pneumonia” and published as an abstract.^2^, ^3^

Both the abstract and this publication appear to present the same data based on an identical study cohort at identical time points (T0‐admission, n = 17; T1‐2 weeks, n = 13, and T2, 30‐days, n = 6); 16 of 17 dogs having shred signs at T0; and median C‐reactive protein (CRP) values of 129 mg/L (24‐267) at T0, 7.7 (3‐32) at T1, and 5.2 (3‐8) at T2 when comparing Table 1 of the EVECCS published abstract and this article in the Journal of Veterinary Internal Medicine.^1^, ^3^ It appears that the authors changed their lung ultrasound protocol from an imprecisely defined 9‐view sliding methodology called “point of care thorax” as described in their abstract^2^, ^3^ to a 9‐view lung ultrasound protocol.^1^ In other words, the authors changed from a nondiscrete sliding protocol over the upper, middle and lower thirds of the thorax as approximately—view 1‐8th intercostal space (ICS), view 2‐5th ICS, view 3‐3rd ICS, view 4‐3rd ICS, view 5‐5th ICS, view 6‐6th ICS, view 7‐6th ICS, view 8‐5th ICS, and view 9‐4th ICS to a discrete intercostal lung examination of the 4th ICS, 6th ICS and 8th ICSs in the upper third, middle third, and lower third of the thorax.

I have concerns about the protocol described in the abstract because the overlays described for a lateral thoracic radiograph are not possible because the forelimb, and its muscular attachments, limit regions of the lung available for ultrasound imaging. Furthermore, the revised protocol with a discrete intercostal lung examination is similarly problematic because the upper and middle third of the 4th ICS and the middle and lower third of the 8th ICS are inaccessible for lung imaging in most dogs because of forelimb interference cranially and imaging over the abdominal cavity caudally. A final point worthy of mention, the recording of lung ultrasound findings in medical records is imprecise with the sliding 9‐views. In my opinion, it is unlikely from their sliding lung ultrasound saved cine clips that the authors could have, post hoc, accurately evaluated a revised protocol from images saved from 2019. Regardless, in my view a change in methodology from that used in the published abstract should have been stated in the methods section of this article.

## CONFLICT OF INTEREST DECLARATION

Dr Lisciandro is the co‐owner of FASTVet.com, a private corporation that provides veterinary ultrasound training to practicing veterinarians. He also teaches ultrasound courses and has received equipment on loan from SonoSite, Sound, scil animal care, Universal Imaging, iVetUltrasound and EI Medical. Dr Lisciandro has licensed educational materials to EI Medical and Veterinary Medical Network and has been a paid consultant for Oncura Partners and Vet Imaging. His wife Stephanie is Medical Director at Oncura Partners. Dr Lisciandro is credited with developing the Vet BLUE technique which is a registered educational service mark to uphold the exam's integrity through proper training.
